# Urban Milk Supply

**Published:** 1905-11-25

**Authors:** 


### The Hospital.

A Journal of

The Medical Sciences and Hospital Administration.

Vol. XXXIX.—No. 1,000. SATURDAY, NOVEMBER 25, 1905.

### Urban Milk Supply.

On more than one recent occasion we have had to take favourable notice of the efforts of sanitarians to direct attention to the quality of the milk supplied to urban populations, and which forms, in most cases, the customary food of urban children. Medical officers of health on various suitable occasions, have proclaimed repeatedly upon the subject; possibly with the result of causing it to be looked upon as one pertaining somewhat more to health officers as such than to the members of the medical profession generally. We should gladly see it regarded in no way as a matter requiring only official cognisance and supervision, but as one coming home not only to every private practitioner, but to every mother or mistress of a household. It cannot be doubted that the last half century has witnessed a very remarkable diminution of the practice of feeding infants from the maternal breast, a corresponding tendency to rely upon artificial foods which generally have their basis in cow's milk, an infantile mortality which remains excessive, and is in some localities appalling, while the mortality at all other ages has steadily diminished, and at least an apparent tendency to physical degeneracy among the children of the industrial classes; as if those who survived the perils of the first year of life had yet not passed through them unscathed, and were growing up in a condition below the standard of vitality proper to their class and necessary for the adequate discharge of their prospective duties in life. If all this be so, it is impossible not to sympathise with the view of the subject taken by Dr. Aitchison Robertson, the Lecturer on Public Health in the School of Medicine of the Royal Colleges, Edinburgh, who, in a paper read before the Medico-Chirurgical Society of that city, declared the time to be come when the question of urban milk should be referred to general medical practitioners, each one of whom is in reality an officer of health, and through whom the public might be educated to a fuller appreciation of the necessity for a more stringent regulation of our supply. Dr. Robertson dwelt mainly upon the conditions existing in Edinburgh itself, but much that he had to say concerning them is of very wide or even general applicability.

The first point to which he directed attention was the reason why so many infants and young children suffer from intestinal tuberculosis; and, while declaring it to be obvious that the tubercle bacillus cannot gain entrance to the system through the intestinal mucous membrane unless the latter be diseased, he declared it to be probable that the bacilli easily penetrate a debilitated and feebly resisting epithelial lining to the intestinal tract, and that this loss of the normal resisting power is due in very many cases to the ingestion of milk which is about to putrefy. Diarrhœa is readily produced in children by any food which is beginning to "turn," and milk, of all organic fluids, is the most prone to undergo decomposition, due to the presence of organisms known generally as dairy bacteria, and which lead to the formation of lactic acid. When freshly drawn from pasture-fed cows milk has either a slight alkaline reaction or it alters the colour of both red and blue test paper; but when drawn from stall-fed cattle it is often slightly acid. This acidity is caused by the presence of the lactic acid bacillus, either in the milk ducts, in the pails, or in the air of the shed. In any case, the lactic acid fermentation soon commences, and progresses with a rapidity dependent upon the way in which the milk is treated and upon the atmospheric conditions. In milk sold as fresh in thirty-one shops in Edinburgh, Dr. Robertson found lactic acid in varying quantities. If lactic acid be given to infants, even in minute quantity, it produces gastric irritation, flatulence, pain, looseness of the bowels, and diarrhœa. If infants are fed upon an organic liquid more or less rich in this acid, and containing, it may be, myriads of other pathogenic and fermentative organisms, the intestinal mucous membrane undergoes severe irritation with resultant diarrhœa, often terminating fatally; and, if tubercle bacilli of bovine origin be introduced into an intestine the mucous lining of which has been thus weakened, the conditions are present for the easy passage of the bacilli into and through the mucosa, with the result that abdominal tuberculosis supervenes.

The presence of tubercle bacilli in the milk is unfortunately only too common an occurrence; and it is one that is greatly promoted by the whole modern system of stall-kept cows; while the presence of putrefactive bacilli is equally provided for by the systematic uncleanlines of urban dairies. The animals are closely confined in a hot and vitiated atmosphere, usually with very imperfect arrangements for the removal of refuse. They are seldom or never effectively groomed, and the hands and clothing of the milkers and attendants are often filthy in the extreme, so that dried particles of the cow's excreta, hairs, epithelial *débris* and organisms readily fall into the open pails in which the milk is received. The only treatment to which the milk is subjected is to pass it through a fine wire strainer, which permits the passage of much solid adventitious matter, and of a still larger amount which undergoes solution. The inspection of the bottom of a jug from which milk had been slowly poured would be a revelation to many of those who are accustomed to swallow the opaque liquid without hesitation. To many practical suggestions for the reform of the evils which he describes, Dr. Robertson adds the expression of his belief that if a crusade against the rearing of infants on cow's milk were carried on, and the immense advantages of maternal nursing insisted upon, the "health conscience" of the population would be aroused. Perhaps the chief want, after all, may be that of example from mothers of high station.

